# Tracking of Vascular Measures From Infancy to Early Childhood: A Cohort Study

**DOI:** 10.1161/JAHA.124.036611

**Published:** 2024-11-04

**Authors:** Toby Mansell, Joel Nuotio, Peter Vuillermin, Anne‐Louise Ponsonby, Deborah A. Lawlor, Kate McCloskey, Markus Juonala, David P. Burgner, the Barwon Infant Study Investigator Group

**Affiliations:** ^1^ Murdoch Children’s Research Institute, Royal Children’s Hospital Parkville Melbourne Australia; ^2^ Department of Paediatrics University of Melbourne Parkville Melbourne Australia; ^3^ Research Centre of Applied and Preventive Cardiovascular Medicine University of Turku Finland; ^4^ Centre for Population Health Research University of Turku and Turku University Hospital Turku Finland; ^5^ Heart Centre Turku University Hospital and University of Turku Finland; ^6^ School of Medicine Deakin University Geelong Australia; ^7^ Barwon Health Geelong Australia; ^8^ Florey Institute of Neuroscience and Mental Health University of Melbourne Melbourne Australia; ^9^ Integrative Epidemiology Unit, University of Bristol UK; ^10^ Population Health Sciences, Bristol Medical School University of Bristol UK; ^11^ Department of Medicine, University of Turku and Division of Medicine Turku University Hospital Turku Finland; ^12^ Department of Paediatrics Monash University Melbourne Australia

**Keywords:** blood pressure, infancy, intima‐media thickness, longitudinal, pulse wave velocity, Epidemiology, Pediatrics, Ultrasound, Blood Pressure

## Abstract

**Background:**

Atherosclerosis develops across the life course, and variation in aortic intima‐media thickness (IMT) is evident from infancy onward, although most early‐life data are cross‐sectional. We investigated whether abdominal aortic IMT at age 6 weeks is associated with vascular measures at 4 years and the relationship of prenatal and perinatal exposures with these measures in early childhood.

**Methods and Results:**

We analyzed data from 518 participants with 6‐week and 4‐year vascular measures from the Barwon Infant Study. Aortic IMT was measured at 6 weeks (mean, 6.1±SD 1.5 weeks) and aortic and carotid IMT, carotid–femoral pulse wave velocity, and blood pressure at 4 years of age (4.3±0.3 years). Associations of early‐life exposures—maternal enteric microbiome, smoking and low‐density lipoprotein cholesterol during pregnancy, birth weight, and gestational age—were also investigated. In the primary model, 6‐week aortic IMT (649±66 μm) was associated with small differences in 4‐year carotid IMT (453±45 μm) (mean difference in carotid IMT per 100 μm higher 6‐week aortic IMT=7.0 μm [95% CI, 0.7–13.3]; *P*=0.03), with no evidence for associations with 4‐year aortic IMT, pulse wave velocity, or blood pressure. Higher birth weight was associated with greater 4‐year aortic IMT, and maternal smoking with higher systolic blood pressure.

**Conclusions:**

Vascular measures do not show strong evidence of tracking between infancy and early childhood. Longitudinal studies with repeated assessment beyond age 4 years would inform optimal timing of early prevention and targets for primordial prevention.

**Nonstandard Abbreviations and Acronyms**
ALSPAC: Avon Longitudinal Study of Parents and Children
IMT: intima‐media thickness
PWV: pulse wave velocity

**Research Perspective**

**What Is New?**

- Aortic intima‐media thickness in infancy was associated with small differences in preschool carotid intima‐media thickness but not aortic intima‐media thickness, pulse wave velocity, or blood pressure.

**What Question Should Be Addressed Next?**

- These findings suggest that antenatal factors related to vascular measures in infancy are not major determinants of vascular structure and function in early childhood.
- Longitudinal studies with repeated vascular measures from early infancy onward and granular data on key exposures from pregnancy and postnatally are needed to better understand which and when vascular measures begin to track into later life.

Cardiovascular disease (CVD), the leading cause of total and premature death worldwide,^1^ has a long preclinical period during childhood and early adulthood before CVD events occur.^2^ Better understanding of early CVD risk would inform the timing and targets for earlier primordial and primary prevention.

Structural and functional preclinical vascular measures suggested to reflect early atherosclerosis are evident in fetuses, infants, and children.^3^ For example, up to 50% of infants have fatty deposits in the aorta.^4^ Previous studies have shown more adverse vascular changes in infants and children at high risk for future CVD, including those born preterm or exposed to maternal CVD risk factors in utero.^5^, ^6^, ^7^ Vascular changes occur at sites where atherosclerotic plaque subsequently develops, and noninvasive vascular measures from adolescence onward predict CVD events.^8^, ^9^

The predictive utility of early‐life vascular measures in long‐term CVD risk assessment is unclear and would necessitate these measures tracking with age. There are now studies that have described longitudinal associations of vascular measures later in childhood, adolescence, and young adulthood,^10^, ^11^, ^12^ but there are no data on tracking of early‐life vascular differences, from infancy to preschool age.

We aimed to investigate whether structural measures of the abdominal aorta at age 6 weeks are associated with structural and functional vascular measures at 4 years. As previous associations of prenatal and perinatal exposures with the 6‐week aortic measures have been reported for this cohort,^13^ we also aimed to estimate associations of prenatal and perinatal exposures with these vascular measures to investigate potential longer‐term effects of these exposures up to age 4 years.

## METHODS

We used data from the Barwon Infant Study, an Australian prebirth cohort (n=1074) recruited using an unselected sampling frame.^14^ In the Barwon Infant Study, vascular measures were obtained at 6 weeks (aortic intima‐media thickness [IMT] only) and age 4 years. We included participants with available 6‐week aortic IMT data and at least one 4‐year vascular measure (n=402 to 472, 518 total; Figure S1; see Table S1 for comparison of these participants with cohort participants without vascular data). The study was approved by the Barwon Health Human Research Ethics Committee (HREC 10/24) and parents provided written informed consent. With the approved ethics for this study, the individual participant data cannot be made freely available online. Interested parties can access the data used in this study upon reasonable request, with approval by the Barwon Infant Study data custodians.

### Vascular Measurements

Vascular measures were obtained using standardized operating procedures, as previously described.^15^

An experienced cardiovascular sonographer trained research staff who used standardized operating procedures and imaging protocols.^16^, ^17^ Images for IMT measurement were obtained using a GE Vivid I ultrasound machine with 10 MHz linear transducer (GE Healthcare, New South Wales, Australia) with simultaneous 3‐lead ECG gating. All IMT images were captured at end‐diastole to reduce physiologic variation during the cardiac cycle.^18^ A longitudinal, straight, unbranched 1‐cm segment of abdominal aorta proximal to the bifurcation was captured between the umbilicus and xiphisternum. Carotid IMT was measured in the right common carotid artery 1 cm proximal to the carotid bulb. Three continuous cineloops of at least 5 cardiac cycles were captured.

Aortic IMT at both time points and 4‐year carotid IMT and diameter were measured using an in‐house semi‐automated edge‐detection software (Coast) programmed in Matlab (R2022b, The Mathworks Inc, Natick, MA) with good reproducibility (intrareader absolute 2‐way mixed‐effect intraclass correlation coefficient=0.93, interreader intraclass correlation coefficient=0.80). Aortic diameter was calculated from ultrasonic caliper measurements.^15^ An average of 3 to 5 measures of carotid‐femoral pulse wave velocity (PWV) and systolic and diastolic blood pressure (BP) were obtained after a 5‐minute rest using the SphygmoCor XCEL device (AtCor Medical Pty, New South Wales, Australia).

### Pre‐/Perinatal Exposures and Potential Confounders

Prenatal and perinatal exposures of interest were selected on the basis of previous studies in Barwon Infant Study^13^ and other studies in the field.^4^, ^6^ Maternal group B streptococcus colonization (a proxy of the maternal vagino‐enteric microbiome),^19^, ^20^ birth weight, and gestational age were each associated with 6‐week aortic IMT in a previous study in the Barwon Infant Study.^13^ In Australia, group B streptococcus screening at 35 to 37 weeks' gestation with a combined low vaginal and anorectal swab is performed to identify infants at risk of group B streptococcus infection.^21^ Maternal group B streptococcus colonization status (yes/no), gestational age (weeks), and birth weight (kg) were extracted from hospital records. As maternal smoking^22^ and maternal hypercholesteremia^6^ have been previously linked to differences in aortic intima‐media thickness or fatty streaks in neonates or children, we also considered maternal smoking during pregnancy and maternal low‐density lipoprotein cholesterol as exposures. Maternal smoking was collected from questionnaire data, considered here as a binary any/none variable with any smoking defined as ≥1 cigarettes reported during any of the 3 trimesters. Maternal low‐density lipoprotein cholesterol (mmol/L) was measured in plasma collected at 28 weeks of pregnancy using the Nightingale Health (Finland) nuclear magnetic resonance metabolomics platform,^23^ as described previously.^24^

Maternal age (years), prepregnancy body mass index (kg/m^2^), education (university graduate education/less than graduate education), and parity (first birth/subsequent birth), collected from questionnaires and hospital records, were identified a priori as potential confounders in effects of pre‐/perinatal exposures on vascular measures.

### Statistical Analysis

Analyses were performed using Stata version 17.0 (StataCorp, College Station, TX). All analyses were adjusted for sex and exact age. Partial Pearson's correlations were calculated between 6‐week aortic IMT and 4‐year vascular measures (aortic IMT, carotid IMT, PWV, systolic BP, and diastolic BP) with bootstrap 95% CIs estimated with 400 replications. To investigate tracking of vascular phenotypes from age 6 weeks to 4 years, linear regression models with 6‐week aortic IMT as the explanatory variable and 4‐year vascular measures as an outcome were used, with statistical significance inferred at a 2‐tailed *P* value <0.05. Participants were included in all models for which they had available data (n=402 to 472 for primary analyses). Estimates are presented per 100‐μm difference in 6‐week aortic IMT. To estimate the effect of pre‐/perinatal exposures on 6‐week aortic IMT and 4‐year vascular measures, linear regression models adjusted for maternal age, prepregnancy body mass index, education, and parity were used.

In secondary analyses, additional model adjustments were explored: (1) adjustment for aortic (and carotid, as appropriate) vessel diameter; (2) adjustment for pre‐/perinatal factors (maternal age, maternal prepregnancy body mass index, maternal education, parity, maternal group B streptococcus colonization, maternal smoking, and maternal low‐density lipoprotein cholesterol, birth weight, and gestational age); and (3) adjustment for vessel diameter and pre‐/perinatal factors.

To explore potential sex differences in the association between 6‐week aortic IMT and 4‐year vascular measures, sex‐stratified models were considered. To test for sex‐interaction effects, models with an interaction term between 6‐week aortic IMT and sex were considered, with statistical significance for the interaction term inferred at a 2‐tailed *P* value <0.1. As with the main models, we also explored sex‐stratified and sex‐interaction models additionally adjusted for (1) aortic (and carotid, as appropriate) vessel diameter, (2) pre‐/perinatal factors, and (3) both vessel diameter and pre‐/perinatal factors.

For the models estimating the effects of pre‐/perinatal exposures on vascular measures, we similarly explored models additionally adjusted for vessel diameter for the relevant vascular measures (6‐week aortic IMT, 4‐year aortic and carotid IMT) as secondary analyses.

## RESULTS

Participant characteristics are shown in Table 1. Participant characteristics stratified by sex are shown in Table S2. There was a weak longitudinal correlation between 6‐week aortic IMT (mean, 649±SD 66 μm) and 4‐year carotid IMT (453±45 μm) (partial Pearson's correlation, *r*=0.10 [95% CI, 0.01–0.20]; *P*=0.03), but not aortic IMT (525±57 μm) (*r*=−0.05 [95% CI, −0.15 to 0.04], *P*=0.28), PWV (3.96±0.44 m/s) (*r*=0.06 [95% CI, −0.02 to 0.15]; *P*=0.18), systolic BP (106.9±8.7 mm Hg) (*r*=0.03 [95% CI, −0.07 to 0.13]; *P*=0.54), nor diastolic BP (64.6±6.9 mm Hg) (*r*=0.03 [95% CI, −0.07 to 0.14]; *P*=0.50). Cross‐sectional correlations between 4‐year vascular measures are shown in Table S3. At 4 years, carotid IMT and systolic BP were correlated (*r*=0.12 [95% CI, 0.02–0.22]; *P*=0.02).

**Table 1:** Characteristics of the Study Participants (n=518)

Characteristic	N	n	%
Sex, female	518	247	48
Pre‐/perinatal factors			
Maternal education, university education	511	304	59
Maternal smoking during pregnancy, any	515	61	12
Maternal group B streptococcus colonization, yes	492	89	18
Parity, first birth	518	225	43

	N	Mean	SD	Range
Maternal age, y	518	31.9	4.3	18.1–48.9
Maternal prepregnancy body mass index, kg/m^2^	479	25.5	5.4	15.6–50.5
Maternal low‐density lipoprotein cholesterol, mmol/L	508	2.19	0.70	0.55–5.36
Birth weight, kg	518	3.59	0.51	1.66–5.41
Gestational age, wks	518	39.7	1.3	33.7–42.0
6‐week time point				
Age, wks	518	6.0	1.5	3.3–18.3
Aortic intima‐media thickness, μm	518	649	66	488–856
Aortic minimum diameter, μm	497	4986	534	3255–6899
4‐year time point				
Age, y	518	4.2	0.3	3.9–5.5
Aortic intima‐media thickness, μm	402	525	57	391–787
Aortic minimum diameter, μm	436	6318	848	4246–11 277
Carotid intima‐media thickness, μm	460	453	45	348–589
Carotid minimum diameter, μm	447	5173	373	4267–6724
Pulse wave velocity, m/s	472	3.96	0.44	2.75–6.9
Systolic blood pressure, mm Hg	434	106.9	8.7	86.0–155.5
Diastolic blood pressure, mm Hg	434	64.6	6.9	43.3–102.0

N indicates the number of participants with available data for each measure. Variables are presented as mean, SD, and range for continuous variables and as number (n) and percentage (%) for binary variables.

Estimated effects of 6‐week aortic IMT on 4‐year vascular measures are shown in Table 2. Overall, in primary models (age‐ and sex‐adjusted) there was little evidence of 6‐week aortic IMT associating with 4‐year vascular measures, with the strongest evidence seen for carotid IMT (mean difference in carotid IMT, 7.0 μm [95% CI, 0.8–13.4] per 100 μm higher 6‐week aortic IMT; *P*=0.03). In models additionally adjusted for vessel diameter, pre‐/perinatal exposures, or both, there were no associations between 6‐week aortic IMT and 4‐year vascular measures (Table 2, Table S4). There was generally no evidence for sex differences (Table S5), although higher 6‐week aortic IMT was associated with lower 4‐year aortic IMT in girls but not boys when adjusted for vessel diameter and pre‐/perinatal exposures (mean 4‐year aortic IMT difference −15.7 μm [95% CI, −27.7 to −3.6] per 100 μm higher 6‐week aortic IMT in girls; mean difference, 1.2 μm [95% CI, −13.3 to 15.7] for boys; *P*‐interaction=0.07).

**Table 2:** Associations Between Aortic Intima‐Media Thickness at 6 Weeks of Age and Vascular Measures At 4 Years

4‐year vascular measure	Age‐ and sex‐adjusted models				Additional adjustment for vessel diameter*				Additional adjustment for pre‐/perinatal factors†			
	MD	95% CI	*P value*	n	MD	95% CI	*P*	n	MD	95% CI	*P value*	n
Aortic IMT, μm	−4.79	−13.46 to 3.87	0.28	402	−6.55	−14.94 to 1.84	0.13	381	−6.55	−16.18 to 3.08	0.18	345
Carotid IMT, μm	7.05	0.76 to 13.35	0.03	460	4.28	−2.25 to 10.80	0.20	430	6.66	−0.35 to 13.68	0.06	391
Pulse wave velocity, m/s	0.042	−0.020 to 0.104	0.18	472	0.032	−0.031 to 0.095	0.32	452	0.038	−0.033 to 0.109	0.30	403
Systolic blood pressure, mm Hg	0.379	−0.845 to 1.602	0.54	434	0.237	−1.021 to 1.494	0.71	417	0.313	−1.029 to 1.656	0.65	363
Diastolic blood pressure, mm Hg	0.334	−0.634 to 1.302	0.50	434	0.046	−0.945 to 1.036	0.93	417	0.325	−0.767 to 1.417	0.56	363

Results presented are MD in vascular outcome at age 4 years per 100‐μm higher 6‐week aortic IMT and the 95% confidence interval from linear regression models. IMT indicates intima‐media thickness; and MD, mean difference.

* Adjusted for age at each time point, sex, 6‐week aortic diameter, and if applicable, 4‐year aortic or carotid diameter.

^†^ Adjusted for age at each time point, sex, maternal age, maternal prepregnancy body mass index, maternal education, parity, maternal group B streptococcus colonization, maternal smoking, and maternal low‐density lipoprotein cholesterol, birth weight, and gestational age.

Estimated effects of pre‐/perinatal exposures on vascular measures are shown in Table 3 and Table S6. Maternal group B streptococcus colonization and birth weight were both associated with higher 6‐week aortic IMT, as previously reported.^13^ At 4 years, birth weight was associated with 4‐year aortic IMT (mean difference, 14.3 μm [95% CI, 2.4–26.3] per 1‐kg higher birth weight; *P*=0.02), and maternal prenatal smoking was associated with systolic BP (mean difference, 4.0 mm Hg [95% CI, 1.3–6.7] compared with children of nonsmokers; *P*=0.004).

**Table 3:** Associations Between Pre‐/Perinatal Exposures and Intima‐Media Thickness at Age 6 Weeks and Vascular Measures at 4 Years

Pre‐/perinatal exposure	6‐week aortic IMT				4‐year aortic IMT				4‐year carotid IMT			
	MD	95% CI	*P* value	n	MD	95% CI	*P* value	n	MD	95% CI	*P* value	n
Maternal group B streptococcus colonization, yes	28.11	12.19 to 44.04	0.0006	446	−10.97	−26.77 to 4.82	0.17	352	1.32	−10.23 to 12.88	0.82	399
Maternal smoking, any	−6.10	−25.10 to 12.90	0.53	469	2.02	−16.68 to 20.73	0.83	370	−7.16	−20.47 to 6.14	0.29	420
Maternal low‐density lipoprotein cholesterol, mmol/L	7.09	−1.50 to 15.69	0.11	465	4.43	−4.30 to 13.15	0.32	365	5.38	−0.78 to 11.55	0.09	416
Birth weight, kg	25.91	13.92 to 37.89	<0.0001	472	14.34	2.42 to 26.77	0.02	372	1.29	−7.30 to 9.89	0.77	423
Gestational age, wks	3.81	−0.97 to 8.59	0.12	472	4.12	−0.42 to 8.67	0.08	372	−2.23	−5.61 to 1.14	0.19	423

	4‐year PWV				4‐year systolic BP				4‐year diastolic BP			
	MD	95% CI	*P* value	n	MD	95% CI	*P* value	n	MD	95% CI	*P* value	n
Maternal group B streptococcus colonization, yes	−0.040	−0.155 to 0.076	0.50	410	−0.144	−2.538 to 2.251	0.91	369	−0.208	−2.145 to 1.728	0.83	369
Maternal smoking, any	−0.045	−0.175 to 0.086	0.50	434	3.983	1.264 to 6.703	0.004	393	1.584	−0.607 to 3.776	0.16	393
Maternal low‐density lipoprotein cholesterol, mmol/L	0.011	−0.050 to 0.071	0.73	429	0.293	−0.936 to 1.523	0.64	389	0.289	−0.694 to 1.271	0.56	389
Birth weight, kg	0.038	−0.048 to 0.123	0.39	436	0.234	−1.515 to 1.984	0.79	395	0.051	−1.351 to 1.453	0.94	395
Gestational age, wks	−0.006	−0.039 to 0.028	0.74	436	−0.026	−0.705 to 0.653	0.94	395	−0.237	−0.781 to 0.306	0.39	395

Results presented are MD in vascular outcome per 1‐unit change in pre‐/perinatal exposure (for continuous exposures) or in the exposed group compared with the nonexposed (for binary exposures) and the 95% CI from linear regression models. Models are adjusted for child age at the relevant time point, sex, maternal age, maternal prepregnancy body mass index, maternal education, and parity. BP indicates blood pressure; IMT, intima‐media thickness; MD, mean difference; and PWV, pulse wave velocity.

## DISCUSSION

This study is the first to investigate the relationship of aortic IMT in infancy and vascular measures in early childhood. There was no evidence for longitudinal associations between infant aortic IMT and subsequent vascular measures of a magnitude that is likely to be clinically relevant. Early life is a period of rapid growth when extensive vascular remodeling occurs,^25^ which may contribute to the absence of evidence of tracking of vascular measures during the first years of life.

Analogous studies in early life are scarce, with longitudinal studies of childhood vascular measures predominantly reporting findings only from midchildhood onward. BP appears to track from infancy onward and is associated with later CVD risk.^26^ In children aged 6 to 8 years, BP, PWV, and retinal microvascular measures were longitudinally correlated with follow‐up vascular measures 4 years later,^10^ and in young school‐age children, carotid artery longitudinal motion (a measure of arterial stiffness) tracked over a 1‐year follow‐up period in a sex‐specific manner.^27^ In the (ALSPAC) Avon Longitudinal Study of Parents and Children birth cohort, a trajectory of high systolic BP from 9 to 17 years of age was associated with higher carotid IMT at 17 years,^11^ and higher systolic BP and diastolic BP from 9 to 24 years were associated with higher carotid IMT and PWV, respectively, at 24 years.^12^ Data from young adults from the Cardiovascular Risk in Young Finns Study have demonstrated moderate tracking for carotid IMT (Pearson's correlation, *r*=0.46 in women, *r*=0.56 in men) in 1809 adults (aged 24–39 years) over a 6‐year period.^28^ While these studies support tracking of vascular measures from midchildhood onward, our results suggest vascular measures in infancy do not track to early childhood.

In our study, birth weight was associated with aortic IMT at both 6 weeks and 4 years of age, but other associations of pre−/perinatal exposures associated with 6‐week aortic IMT were no longer evident at 4 years. Previous studies investigating associations of pre−/perinatal exposures with offspring vascular measures have largely considered vascular measures at a single time point, with most studies investigating blood pressure as the outcome, and not other cardiovascular measures, such as IMT or PWV. Exposures including preeclampsia,^29^, ^30^ gestational diabetes,^31^ preterm birth,^32^ and very low birth weight^33^ have been associated with higher offspring blood pressure in childhood or young adulthood. Less evidence exists for associations with the other vascular measures considered in this study: preeclampsia and hypertension in pregnancy have been associated with higher aortic IMT in neonates^34^ and carotid IMT in young adults^35^ but not with PWV in children.^30^ Exposure to maternal smoking during pregnancy has also been linked to higher aortic IMT in neonates,^22^ and preterm birth to higher aortic IMT in preschool children^36^ and higher carotid IMT in adolescents.^37^ In our study of early life vascular measures, we have focused on pre‐/perinatal exposures previously associated with 6‐week aortic IMT measures in this cohort,^13^ and exposures that have been linked to vascular measures early in life in other studies, including maternal smoking^22^ and cholesterol.^6^ The cohort in our study excluded preterm births (<32 completed weeks' gestation), which may contribute to the lack of evidence for persistent effects of gestational age on vascular measures in contrast to previous studies of preterm births.

In adults, both higher carotid IMT and PWV are associated with higher CVD risk.^8^, ^18^ Noninvasive assessment of vascular structure and function in childhood has the potential to enhance the evaluation of CVD before irreversible vascular damage.^2^ For example, a life‐course approach using the Special Turku Coronary Risk Factor Intervention Project cohort indicated that accumulation of risk exposure to BP levels at all life stages contributed to adulthood carotid IMT.^38^ In addition, exposure to CVD risk factors such as smoking is already associated with differences in carotid IMT in youth,^39^ supporting the importance in measuring IMT earlier in the life course. However, there is a lack of longitudinal data on the relationship between childhood IMT, PWV, and adult CVD risk. Importantly, the few studies of cardiovascular measures in infancy are largely cross‐sectional.^5^, ^6^, ^7^

Strengths of our study include the novelty of the research question and findings, the large population‐derived sample, standardized measurement of far‐wall IMT at end‐diastole, multiple frames, and semi‐automated edge‐detection software to measure aortic and carotid IMT, which all improve accuracy and reproducibility.^3^, ^15^ Limitations include lack of ethnic diversity, limiting generalizability of our findings. The vascular measures were performed by trained research staff rather than specialist cardiovascular sonographers, but reproducibility of all measures was excellent.^15^ The complete case analysis approach may be affected by potential selection bias from cohort attrition between the two time points in this study.

## CONCLUSIONS

In this large, longitudinal population‐derived cohort, aortic IMT in infancy was not associated with likely clinically relevant differences in vascular measures at age 4 years. Longitudinal studies from early life with serial measures over a wider age range would identify the age at which tracking of vascular measures becomes evident and inform the timing and assessment of targeted primordial and primary CVD prevention.

## APPENDIX

Barwon Infant Study Investigator Group: Murdoch Children's Research Institute, Royal Children's Hospital, Parkville, Melbourne, Australia: John Carlin, Mimi L. K. Tang, Richard Saffery, Sarath Ranganathan, Martin O'Hely. Department of Pediatrics, University of Melbourne, Parkville, Australia: John Carlin, Mimi L. K. Tang, Richard Saffery, Sarath Ranganathan. Royal Children's Hospital, Melbourne, Australia: Mimi L. K. Tang, Sarath Ranganathan. School of Medicine, Faculty of Health, Deakin University, Geelong, Australia: Martin O'Hely, Fiona Collier, Lawrence Gray. The University of Queensland Child Health Research Centre, South Brisbane, Australia: Peter Sly.

## Sources of Funding

The establishment work and infrastructure for the Barwon Infant Study was provided by the Murdoch Children's Research Institute, Deakin University, and Barwon Health. Subsequent funding was secured from the National Health and Medical Research Council of Australia, the Jack Brockhoff Foundation, the Scobie Trust, the Shane O'Brien Memorial Asthma Foundation, the Our Women's Our Children's Fund‐Raising Committee at Barwon Health, The Shepherd Foundation, the Rotary Club of Geelong, the Ilhan Food Allergy Foundation, GMHBA Limited, the Percy Baxter Charitable Trust managed by Perpetual Trustees, and the Minderoo Foundation. In‐kind support was provided by the Cotton On Foundation, and CreativeForce. Research at Murdoch Children's Research Institute is supported by the Victorian Government's Operational Infrastructure Support Program. This work is also supported by project grants from the National Health and Medical Research Council of Australia (GTN1030701 and GTN1164212). Dr Nuotio is supported by Juho Vainio Foundation, Turku University Foundation, Yrjö Jahnsson Foundation, and Finnish Foundation for Cardiovascular Research. Dr Burgner is supported by an Investigator Grant from the National Health and Medical Research Council of Australia (GTN1175744). Dr Mansell is supported by an ECR Fellowship provided by the Murdoch Children's Research Institute. Dr Lawlor's contributions were supported by the British Heart Foundation (AA/18/1/34219 and CH/F/20/90003), the University of Bristol, and the UK Medical Research Council (MC_UU_00032/05). The funding bodies did not play any role in the study. The views expressed in this paper are those of the authors and not necessarily any funders or anyone acknowledged.

## Disclosures

None.

## Supporting information

Tables S1–S6
Figure S1
