# Supplementary material for: Tracking of Vascular Measures From Infancy to Early Childhood: A Cohort Study
Source: J Am Heart Assoc. 2024 Nov 4;13(21):e036611. doi: 10.1161/JAHA.124.036611 (PMC11935727; doi:10.1161/JAHA.124.036611)
Supplement: Supplementary file 1 — Tables S1–S6 Figure S1 [file JAH3-13-e036611-s001.pdf]

SUPPLEMENTAL MATERIAL

Table S1. Comparison of baseline characteristics between Barwon Infant Study participants that were included in this study (n=518) compared to those without the required vascular data (n=556).

	**Participants in this study (n=518)**				**BIS inception cohort not included in this study (n=556)**				**p-value for difference[1]**
**Characteristic**	**N**	**n**	**%**		**N**	**n**	**%**		
Sex, female	518	247	48%		556	272	49%		0.69
Pre-/perinatal factors									
Maternal education, university education	511	304	59%		544	242	44%		<0.001
Maternal smoking during pregnancy, any	515	61	12%		546	108	20%		<0.001
Maternal group B streptococcus colonization, yes	492	89	18%		512	124	24%		0.02
Parity, first birth	518	225	43%		556	255	46%		0.42
	**N**	**Mean**	**SD**	**Range**	**N**	**Mean**	**SD**	**Range**	
Maternal age (years)	518	31.9	4.3	18.1 - 48.9	556	30.8	5.2	17.4 - 44.9	0.002
Maternal pre-pregnancy BMI (kg/m^2)	479	25.5	5.4	15.6 - 50.5	448	25.3	5.5	17.0 - 51.8	0.68
Maternal LDL cholesterol (mmol/L)	508	2.19	0.70	0.55 - 5.36	533	2.19	0.63	0.48 - 5.16	0.95
Birth weight (kg)	518	3.59	0.51	1.66 - 5.41	556	3.46	0.52	1.40 - 4.84	<0.001
Gestational age (weeks)	518	39.7	1.3	33.7 - 42.0	556	39.3	1.7	32.0 - 42.0	<0.001

N indicates the number of participants with available data for each measure. Variables are presented as mean, SD, and range for continuous variables and as number (n) and percentage (%) for binary variables.

[1]Two-tailed p-value for testing the null hypothesis of no true difference between the two groups. P-values for binary characteristics are from chi-squared tests, p-values for continuous characteristics are from Student's t-tests.

Table S2. Characteristics of the study participants (n=518), stratified by sex.

Characteristic	Girls (n=247)				Boys (n=271)			
Pre-/perinatal factors	N	n	%		N	n	%	
Maternal education, university education	244	147	60%		267	157	59%	
Maternal smoking during pregnancy, any	247	28	11%		268	33	12%	
Maternal group B streptococcus colonization, yes	235	40	17%		257	49	19%	
Parity, first birth	247	100	40%		271	125	46%	
	**N**	**Mean**	**SD**	**Range**	**N**	**Mean**	**SD**	**Range**
Maternal age (years)	247	31.9	4.2	18.1 - 48.9	271	31.9	4.3	18.1 - 44.3
Maternal pre-pregnancy BMI (kg/m^2)	227	25.2	5.4	15.6 - 49.9	252	25.7	5.4	17.4 - 50.5
Maternal LDL cholesterol (mmol/L)	245	2.18	0.72	0.55 - 5.36	263	2.21	0.68	0.58 - 4.67
Birth weight (kg)	247	3.53	0.49	1.66 - 4.89	271	3.65	0.52	2.13 - 5.41
Gestational age (weeks)	247	39.6	1.3	33.7 - 41.9	271	39.7	1.2	34.7 - 42.0
6-week time point								
Age (weeks)	247	6.0	1.3	3.3 - 10.3	271	6.1	1.6	3.4 - 18.3
Aortic intima-media thickness (µm)	247	651	67	488 - 856	271	648	65	497 - 854
Aortic minimum diameter (µm)	241	4842	485	3255 - 6899	256	5123	543	3785 - 6533
4-year time point								
Age (years)	247	4.2	0.3	3.9 - 5.5	271	4.2	0.3	3.9 - 5.5
Aortic intima-media thickness (µm)	199	525	54	409 - 731	203	525	60	391 - 787
Aortic minimum diameter (µm)	217	6106	823	4246 - 9665	219	6528	823	4475 - 11277
Carotid intima-media thickness (µm)	221	450	46	350 - 589	239	455	44	348 - 580
Carotid minimum diameter (µm)	212	5094	356	4267 - 6724	235	5246	374	4320 - 6517
Pulse wave velocity (m/s)	229	3.94	0.41	3.07 - 5.20	243	3.97	0.47	2.75 - 6.90
Systolic blood pressure (mmHg)	213	106.3	8.5	86.0 - 136.5	221	107.6	8.9	88.0 - 155.5
Diastolic blood pressure (mmHg)	213	64.0	6.2	47.0 - 91.5	221	65.1	7.4	43.3 - 102

N indicates the number of participants with available data for each measure. Variables are presented as mean, SD, and range for continuous variables and as number (n) and percentage (%) for binary variables.

Table S3. Cross-sectional correlations of 4-year vascular measures.

4-year vascular measure	Aortic IMT			Carotid IMT			PWV			Systolic BP		
	**r**	**95% CI**	**p**	**r**	**95% CI**	**p**	**r**	**95% CI**	**p**	**r**	**95% CI**	**p**
Carotid IMT	0.029	−0.070, 0.128	0.577	-	-	-	-	-	-	-	-	-
PWV	0.063	−0.043, 0.169	0.224	−0.011	−0.110, 0.089	0.828	-	-	-	-	-	-
Systolic BP	0.090	−0.018, 0.198	0.093	0.119	0.019, 0.220	0.018	−0.015	−0.106, 0.076	0.763	-	-	-
Diastolic BP	0.099	−0.013, 0.211	0.064	−0.020	−0.117, 0.077	0.689	0.085	−0.006, 0.176	0.086	0.728	0.670, 0.786	<0.0001

Correlations are partial Pearson's correlations (r) between the two vascular measures, adjusted for age and sex.

95% confidence intervals are normal bootstrap confidence intervals from 400 replications.

Table S4. Associations between aortic intima-media thickness at 6 weeks of age and vascular measures at 4 years (models additionally adjusted for both vessel diameter and pre-/perinatal exposure).

4-year vascular measure	MD	95% CI	p	n
Aortic IMT (µm)	−7.349	−16.549, 1.85	0.117	326
Carotid IMT (µm)	3.639	−3.526, 10.803	0.319	367
Pulse wave velocity (m/s)	0.034	−0.039, 0.106	0.363	385
Systolic blood pressure (mmHg)	0.090	−1.283, 1.463	0.898	348
Diastolic blood pressure (mmHg)	−0.002	−1.109, 1.106	0.998	348

Results presented are mean difference (MD) in vascular outcome at 4 years of age per 100 µm higher 6-week aortic intima-media thickness and the 95% confidence interval from linear regression models. IMT: intima-media thickness.

Models are adjusted for age at each time point, sex, 6-week aortic diameter (and if applicable, 4-year aortic or carotid diameter), maternal age, maternal pre-pregnancy BMI, maternal education, parity, maternal group B streptococcus colonization, maternal smoking, and maternal LDL cholesterol, birth weight and gestational age.

Table S5. Associations between aortic intima-media thickness at 6 weeks of age and vascular measures at 4 years, stratified by sex.

4-year vascular measure	Girls only				Boys only				p-value for sex-interaction[1]
	MD	95% CI	p	n	MD	95% CI	p	n	
Primary model[2]									
Aortic IMT (µm)	−8.814	−20.336, 2.707	0.133	199	0.267	−12.823, 13.357	0.968	203	0.271
Carotid IMT (µm)	8.773	−0.592, 18.138	0.066	221	5.100	−3.445, 13.645	0.241	239	0.565
Pulse wave velocity (m/s)	0.023	−0.057, 0.103	0.577	229	0.060	−0.035, 0.155	0.212	243	0.593
Systolic blood pressure (mmHg)	−0.095	−1.758, 1.568	0.911	213	0.895	−0.929, 2.719	0.335	221	0.439
Diastolic blood pressure (mmHg)	−0.418	−1.63, 0.794	0.498	213	1.175	−0.352, 2.702	0.131	221	0.112
Additional adjustment for vessel diameter[3]									
Aortic IMT (µm)	−12.169	−23.198, −1.141	0.031	190	−0.248	−13.083, 12.587	0.970	191	0.146
Carotid IMT (µm)	6.168	−3.83, 16.166	0.225	207	2.223	−6.48, 10.925	0.615	223	0.517
Pulse wave velocity (m/s)	0.028	−0.053, 0.11	0.495	223	0.037	−0.06, 0.134	0.456	229	0.804
Systolic blood pressure (mmHg)	0.097	−1.578, 1.772	0.909	208	0.360	−1.567, 2.286	0.713	209	0.889
Diastolic blood pressure (mmHg)	−0.459	−1.683, 0.765	0.461	208	0.631	−0.971, 2.234	0.438	209	0.319
Additional adjustment for pre-/perinatal factors[4]									
Aortic IMT (µm)	−14.798	−27.772, −1.825	0.026	172	0.717	−14.036, 15.47	0.924	173	0.144
Carotid IMT (µm)	4.715	−5.239, 14.669	0.351	193	7.303	−2.907, 17.512	0.160	198	0.758
Pulse wave velocity (m/s)	0.040	−0.053, 0.133	0.397	197	0.065	−0.047, 0.176	0.252	206	0.640
Systolic blood pressure (mmHg)	0.196	−1.526, 1.918	0.822	178	0.520	−1.684, 2.723	0.642	185	0.952
Diastolic blood pressure (mmHg)	−0.554	−1.807, 0.699	0.384	178	1.418	−0.466, 3.303	0.139	185	0.118
Additional adjustment for both vessel diameter and pre-/perinatal factors[5]									
Aortic IMT (µm)	−15.657	−27.722, −3.592	0.011	164	1.201	−13.274, 15.675	0.870	162	0.072
Carotid IMT (µm)	2.126	−8.439, 12.691	0.692	182	3.891	−6.274, 14.056	0.451	185	0.580
Pulse wave velocity (m/s)	0.045	−0.049, 0.138	0.348	192	0.049	−0.065, 0.163	0.399	193	0.904

Systolic blood pressure (mmHg)	0.352	−1.389, 2.092	0.690	174	−0.115	−2.418, 2.188	0.922	174	0.554
Diastolic blood pressure (mmHg)	−0.543	−1.812, 0.727	0.400	174	0.756	−1.213, 2.725	0.450	174	0.330

Results presented are mean difference (MD) in vascular outcome at 4 years of age per 100 μm higher 6-week aortic intima-media thickness and the 95% confidence interval from linear regression models. IMT: intima-media thickness.

[1]P-value for interaction term between sex and 6-week aortic intima-media thickness in a model containing all participants.

[2]Models adjusted for age at each time point.

[3]Models adjusted for age at each time point, and 6-week aortic diameter (and if applicable, 4-year aortic or carotid diameter).

[4]Models adjusted for age at each time point, maternal age, maternal pre-pregnancy BMI, maternal education, parity, maternal group B streptococcus colonization, maternal smoking, and maternal LDL cholesterol, birth weight and gestational age.

[5]Models adjusted for age at each time point, 6-week aortic diameter (and if applicable, 4-year aortic or carotid diameter), maternal age, maternal pre-pregnancy BMI, maternal education, parity, maternal group B streptococcus colonization, maternal smoking, and maternal LDL cholesterol, birth weight and gestational age.

Table S6. Associations between pre-/perinatal exposures and aortic intima-media thickness at 6 weeks of age and aortic and carotid intima-media thickness at 4 years, in models additionally adjusted for vessel diameter.

Pre-/perinatal exposure	6-week aortic IMT				4-year aortic IMT				4-year carotid IMT			
	**MD**	**95% CI**	**p**	**n**	**MD**	**95% CI**	**p**	**n**	**MD**	**95% CI**	**p**	**n**
Maternal Group B streptococcus colonization (yes)	25.985	9.672, 42.298	0.002	427	−12.794	−27.745, 2.157	0.093	345	−0.131	−11.489, 11.227	0.982	390
Maternal smoking (any)	−9.761	−29.21, 9.688	0.324	449	1.927	−15.844, 19.699	0.831	363	−4.975	−18.148, 8.199	0.458	411
Maternal LDL cholesterol (mmol/L)	7.829	−0.939, 16.597	0.080	445	4.092	−4.246, 12.43	0.335	358	4.809	−1.289, 10.908	0.122	407
Birth weight (kg)	0.025	0.012, 0.038	<0.001	452	0.009	−0.002, 0.021	0.123	365	−0.004	−0.012, 0.005	0.410	414
Gestational age (weeks)	0.407	−0.32, 1.135	0.272	452	0.482	−0.135, 1.099	0.125	365	−0.427	−0.903, 0.049	0.079	414

Results presented are mean difference (MD) in vascular outcome per 1 unit change in pre-/perinatal exposure (for continuous exposures) or in the exposed group compared to the non-exposed (for binary exposures) and the 95% confidence interval from linear regression models. BP: blood pressure. IMT: intima-media thickness. PWV: pulse wave velocity.

Models are adjusted for child age at the relevant time point, sex, the relevant vessel diameter, maternal age, maternal pre-pregnancy BMI, maternal education, and parity.

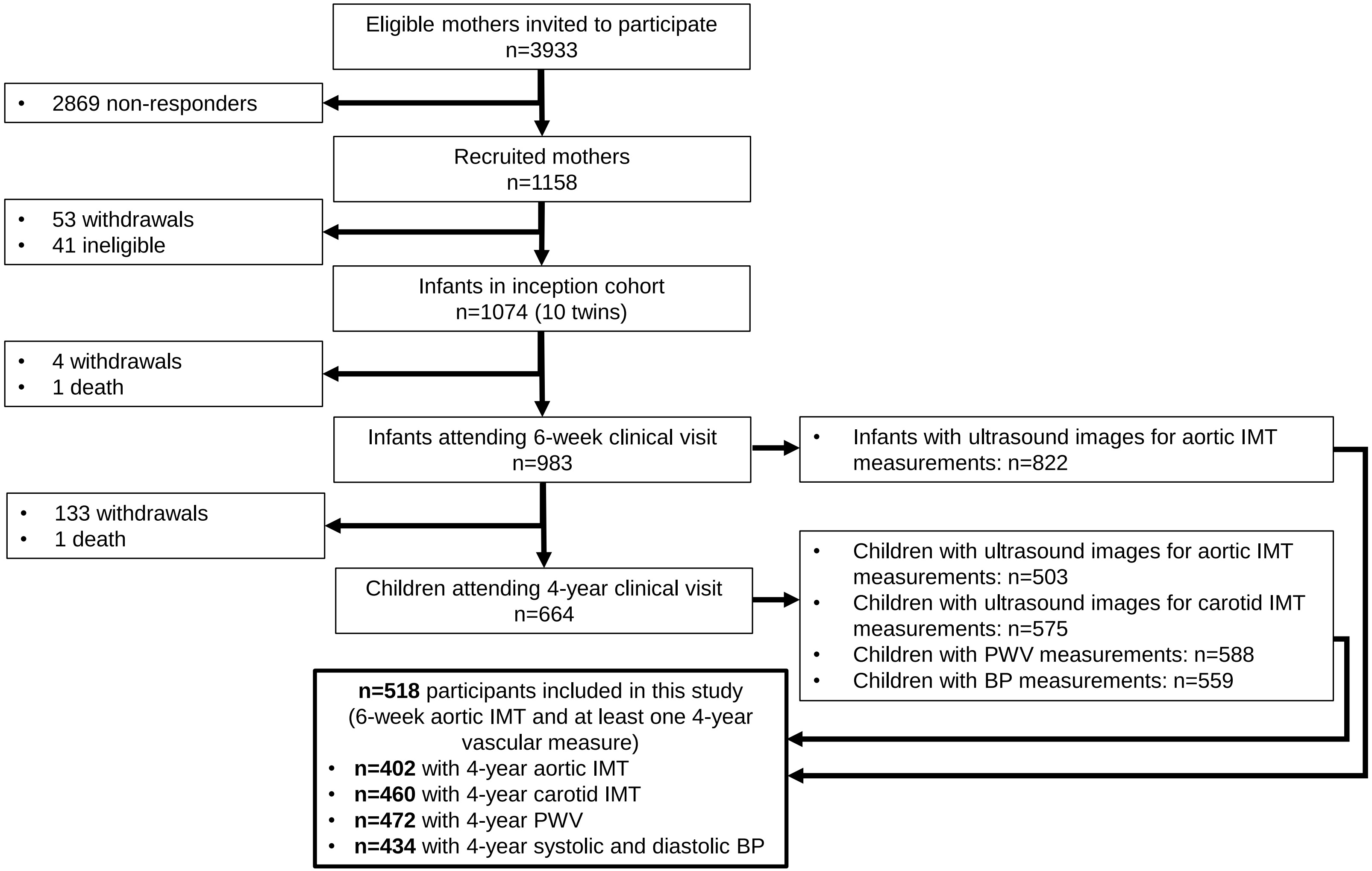

Figure S1. Barwon Infant Study participant flowchart for this study. All participants with 6-week aIMT and any 4-year vascular measures were included in this study (thick outlined box). Comparisons of baseline characteristics between those included in this study and those from the inception cohort not included in this study are shown in **Table S1**. BP: blood pressure. IMT: intima-media thickness. PWV: pulse wave velocity.
